# Paul Gerson Unna: A Medical Pioneer in Dermatopathology

**DOI:** 10.7759/cureus.84288

**Published:** 2025-05-17

**Authors:** Ryan Nguyen, Nadiya A Persaud, Alexandra Franco Garcia

**Affiliations:** 1 Medicine, Orlando College of Osteopathic Medicine, Winter Garden, USA; 2 Research, Orlando College of Osteopathic Medicine, Winter Garden, USA; 3 Infectious Disease, Orlando College of Osteopathic Medicine, Winter Garden, USA

**Keywords:** -dermatopathology, histopathology, historical vignette, medical innovation, seborrheic dermatitis

## Abstract

Paul Gerson Unna, a distinguished dermatologist in Central Europe, created great innovative discoveries. His meticulous research, when combining both skin anatomy and histology, showcased his productivity in dermatology. His initial work on histopathology significantly enhanced the appeal of dermatology to many aspiring professionals. The books that he published not only paved the way to new dermatology methods, such as the Unna boot, but also helped discover new diseases that are associated with pathogens. Although the Unna boot is often noted as a significant innovation, it is just a small part of his extensive contributions to educational research and therapeutic practices in dermatology.

## Introduction and background

**Figure 1:**
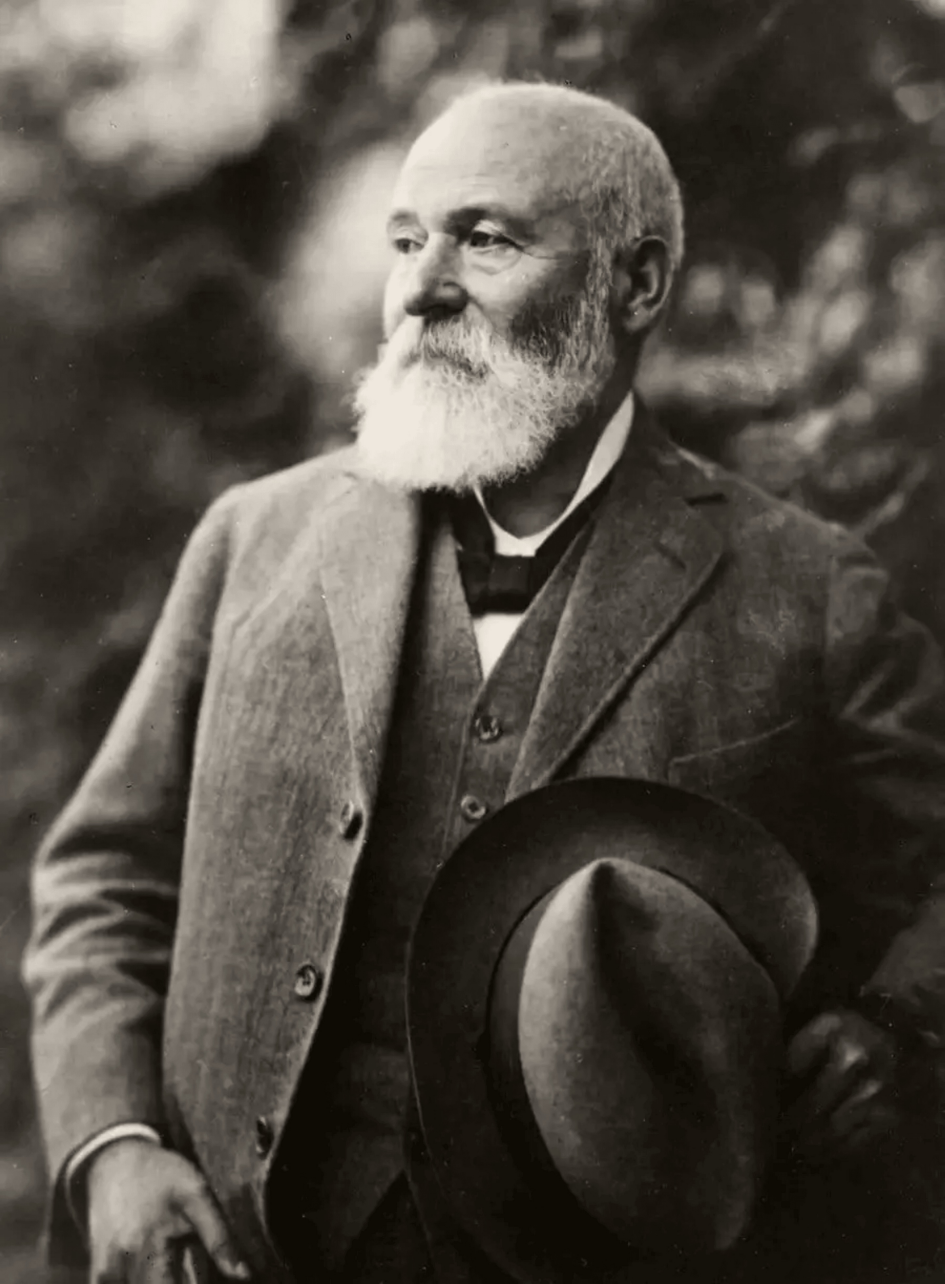
Portrait of Dr. Paul Gerson Unna. Image source: beiersdorf.com available at https://www.beiersdorf.com/about-us/our-history/personalities/paul-gerson-unna [4]. Image available via license: Creative Commons Attribution-NonCommercial-NoDerivatives 4.0 International (https://creativecommons.org/licenses/by-nc-nd/4.0/)

Paul Gerson Unna (Figure 1), a pioneering figure in dermatopathology, was born on September 8, 1850, in Hamburg, Germany. His father, Moritz Adolph Unna, was a well-respected physician, while his mother, Ida Gerson, came from a family with a 200-year tradition of medical practitioners. Unna's older sister, Julie de Boor, achieved recognition as a portrait artist. He attended the Gelehrtenschule des Johanneums in Hamburg and, at 20, added his middle name "Gerson" to honor his grandfather. Unna started his medical education at the University of Heidelberg but paused to serve in the Franco-Prussian War, where he sustained a severe thigh injury from a rifle. Afterward, he resumed his studies in Heidelberg in 1871 and later at the University of Leipzig. Under the mentorship of Professor Heinrich Wilhelm Waldeyer, known for his contributions to anatomy, Unna earned his doctorate in Strasbourg. He trained in dermatology in Vienna with influential figures like Ferdinand von Hebra, Moritz Kaposi, and Heinrich Auspitz. Unna had four children; three pursued careers in dermatology, while the fourth became a pharmacist. Additionally, he was an avid music lover, hosting weekly concerts where he showcased his talents as a cellist [1-3].

Sadly, Unna passed away on January 29, 1929, just four days after revising the manuscript for a brief autobiography, at the age of 78 due to influenza. In recognition of his legacy, the Paul Gerson Unna Academy was established in Germany in 2016, offering a range of practical continuing education courses that enhance knowledge exchange [1-3].

## Review

Introduction to dermatopathology: Unna’s boot

Through his dedication and time to research on dermatology, he was able to combine both dermatology and pathology concepts. Unna collaborated with pharmacists to enhance dermatological formulations and developed a unique gauze bandage in 1885, designed for treating varicose ulcers created by pathogens. This bandage provided cooling and soothing effects with a blend of 15% zinc oxide in a glycerin and gelatin base, offering an alternative to the uncomfortable compression bandages of his time. His technique quickly gained recognition for its effectiveness and straightforward application, and he later presented an improved version at the Third International Congress of Dermatology in 1896, which gained widespread acceptance. Although the use of such bandages declined over time, modified versions are still effective for treating various skin conditions. The "Unna boot" remains a lasting tribute to his contributions and efforts in the field of dermatology [3].

Discovery of seborrheic dermatitis

In 1886, during the International Congress of Medicine in Washington, Paul Gerson Unna delivered a pivotal lecture titled “Seborrhoeal eczema,” which was later published in 1887. In this lecture, he differentiated seborrheic eczema from other forms of eczema, identifying the scalp as the initial site of the disease. Unna characterized the condition by three main features: desquamation (dandruff), crusting (dry scalp), and a moist nature (chronic scalp eczema). He described the lesions as rounded and oval, often merging into serpiginous shapes with distinct borders and yellowish scales. Unna noted that these lesions typically appeared in areas rich in sebaceous glands, linking seborrhea to sebaceous secretion, which he termed "seborrhea sicca." He later proposed a histopathological perspective, identifying the spongiotic state of the epidermis as significant in the disease's manifestation. Seborrheic dermatitis is recognized as a chronic inflammatory condition with a variable incidence across different populations. Despite its initial description in 1887, the understanding of its clinical forms remains incomplete till this day [5-6]. Unna’s pioneering work in identifying the histological features of seborrheic dermatitis laid the groundwork for modern understanding of the disease. His ability to connect clinical symptoms with underlying histopathology has influenced current diagnostic and therapeutic approaches to inflammatory skin conditions. Today, seborrheic dermatitis is understood as a chronic inflammatory disease, and Unna’s insights into the spongiotic nature of the epidermis continue to shape the study of its pathophysiology. Despite significant advances, some clinical forms of seborrheic dermatitis remain elusive, reflecting the enduring relevance of Unna’s initial research in guiding ongoing investigations in dermatology [6].

First publication: Histopathology of the Skin Diseases

In 1894, he published "Histopathology of the Skin Diseases" (*Die Histopathologie der Hautkrankheiten*), a foundational work in dermatopathology, a significant work that emerged from dedicated research into the anatomical and pathological findings of the time, alongside innovative ideas that challenged existing beliefs. This book became an insightful encyclopedia for those who are entering the field of dermatology. Notably, it included detailed pathological insights, clinical observations, and therapeutic guidance that could further help people today on dermatology cases. In his book, he introduced new staining techniques that improved the identification of skin structures and cellular components. His analysis encompassed multiple conditions, including chickenpox and zoster, as well as a thorough investigation of leprosy, for which he developed specific staining methods for the causative bacillus. Unna's explorations extended to a variety of skin disorders, which marked a monumental contribution to the classification and understanding of dermatological diseases [4].

Leaving a legacy

Unna left multiple marks in his life that are further used in new cases for future dermatologists. Firstly, he diagnosed a hyperactive keratin syndrome with his colleague Arthur Thost, and it was named the “Unna-Thost Syndrome”. It is characterized as a diffuse non-epidermolytic palmoplantar keratoderma. It is described as a symmetrical keratoderma that often appears scaly and dry that affects the entire surface of the palms. With the help of Vorner, they helped diagnose a family in 1901 with this syndrome and found histological findings of hyperkeratosis. They examined the family pedigree and confidently determined that it is an autosomal dominant trait. Unna's groundbreaking discovery not only marked a significant achievement but also paved the way for future dermatologists in diagnosing emerging diseases that relate to genetics and histology [7]. Unna also investigated the influence of environmental factors on skin pathology, offering some of the earliest observations on the effects of ultraviolet (UV) radiation. He specifically noted the characteristic skin changes in sailors resulting from chronic sun exposure, a phenomenon he documented in his writings [8]. This pioneering work laid the groundwork for later research into the relationship between UV radiation and skin cancer, significantly contributing to the emerging field of preventive dermatology. In addition, Unna pioneered the description of several dermatological conditions, expanding the lexicon of dermatopathology with terms still in use today. One notable condition he described was “acanthosis nigricans” in 1889, a skin disorder associated with pigmentation and sometimes indicative of underlying malignancies [9]. Furthermore, he introduced the term “cutis verticis gyrata,” referring to a rare scalp condition, in 1907, demonstrating his keen observational capabilities and ability to categorize dermatological phenomena systematically [10]. His work helped in transitioning dermatology into a more organized and scientifically grounded discipline.

Debates and conflicts

Unna's doctoral research, conducted under Professor Waldeyer's supervision, focused on the histology and physiology of epidermis and was published in 1876. His thesis, titled “The Development and Anatomy of the Human Skin and Its Appendages”, introduced a range of innovative ideas and concepts. These original and sometimes controversial proposals faced significant criticism from the German pathologist Friedrich Daniel von Recklinghausen. Unna revised the work multiple times before it was ultimately accepted and published.

In 1884, Unna met the prominent dermatologist Hansen at a medical congress in Copenhagen and joined him on visits to various Norwegian leprosy hospitals. His growing interest in leprosy led to in-depth studies of its pathology and treatment, drawing him into a prolonged and contentious debate with Neisser and his followers that spanned over twenty-five years. Neisser argued that the large polymorphonuclear cells filled with bacilli were specific lepra cells, while Unna contended that they were merely clusters of bacilli surrounded by their own substance, with nuclei belonging to nearby connective tissue cells [2].

## Conclusions

Paul Gerson Unna’s groundbreaking shift from macroscopic observation to microscopic examination revolutionized dermatology and established him as a pioneer in dermatopathology. His integration of histology into clinical practice laid the foundation for modern dermatological science. Although he was a prolific worker and talented writer, he had faced many controversial battles with his research. His determination and dedication on combining dermatology and pathology had escalated a monumental discovery for the field of medicine. The field of dermatology has reached its current advanced status largely thanks to the dedication, passion, and commitment of this memorable pioneer for every dermatologist, Paul Gerson Unna.
